# Romantic bias in judging the attractiveness of faces wearing masks

**DOI:** 10.1177/20416695241287486

**Published:** 2024-10-09

**Authors:** Machi Sugai, Fumiya Yonemitsu, Atsunori Ariga

**Affiliations:** 83546 Chuo University, Hachioji, Japan; 47745 Shibaura Institute of Technology, Saitama, Japan; 83546 Chuo University, Hachioji, Japan

**Keywords:** facial attractiveness, gender difference, sanitary mask, social relationship

## Abstract

The spread of COVID-19 has drastically increased the number of people wearing masks in public areas and the opportunities to evaluate others’ faces based on limited information. This study investigates the cognitive bias in judging the attractiveness of faces partially hidden by sanitary masks. Experiment 1 revealed that men rated women's faces as more attractive when wearing masks, specifically in the context of rating women as romantic partners; however, this mask bias was absent when men rated women as friends. On the other hand, women did not show the mask bias irrespective of the assumed social relationship. Experiment 2 demonstrated that the mask bias among elderly men was less affected by the assumed social relationship (or the possibility of reproduction), compared to young men, though they showed the bias itself. These results suggest that the cognitive strategies related to reproduction underlie the attractiveness judgment of the partial faces.

One of the authors often falls in love with passersby on the street based on the initial visual impressions of their appearance. However (unfortunately), judging the attractiveness of a face's frontal view is rare; in most cases, one judges others’ faces from non-frontal, partial views. Previous research has demonstrated positive correlations between facial attractiveness across frontal, three-quarters, and profile views of the same individuals (Rule et al., 2009). Furthermore, facial attractiveness based on back views was positively correlated with that based on frontal views, even though faces observed from the back were completely invisible (Yonemura et al., 2013). Interestingly, facial attractiveness judged from the back has been consistently overestimated or biased toward positivity (a phenomenon called *back-view bias*), which suggests some sort of cognitive bias or processing strategy in perceiving the partial views of others’ faces. Clarifying not only the correlation of face perception between frontal and partial views but also what underlies the bias in partial views is important to understand the perception of partial faces, which was the focus of this study.

Previously, our colleagues (Ichimura et al., 2021) focused on gender differences in back-view bias, in which male participants showed greater bias in evaluating female faces, whereas female participants showed less bias in evaluating male faces. They hypothesized that such gender differences would reflect mate preference strategies. Facial attractiveness of the opposite gender is an indicator of mate value (e.g., reproductive potential; Gangestad & Scheyd, 2005; Thornhill & Gangestad, 1999). Because the reproductive cost of men is lower than that of women (e.g., Bateman, 1948), a suitable strategy for men is to seek more reproductive opportunities by focusing on women's physical attractiveness (e.g., sexually dimorphic feminine features; Perrett et al., 1998). The overestimation of female facial attractiveness (or greater back-view bias) would drive men to approach women by minimizing Type-II errors (false-negative errors or misses) in error management theory (Haselton & Buss, 2000). On the other hand, women are motivated to minimize Type-I errors (false-positive errors or false alarms) due to prolonged investment in reproduction efforts (e.g., gestation and breastfeeding); as a result, their strategy is to be cautious, focusing on not only physical but also non-physical attractiveness (e.g., good financial prospects) linked to resources for producing and supporting offspring. In summary, men value physical attractiveness more than women do, and they tend to overestimate women's invisible facial attractiveness by seeing them as romantic partners (i.e., potential mates) by default. In contrast, women do not care about physical attractiveness as males do and do not need to overestimate their invisible facial attractiveness.

To test this hypothesis, Ichimura et al. (2021) manipulated the assumed social relationship between raters (participants) and rated models (stimuli) by the instruction. They demonstrated that back-view bias decreased when male participants were explicitly instructed to rate the front/back view of a woman as a friend, although greater bias remained when they rated a woman as a romantic partner; female participants consistently showed less bias irrespective of the social relationship. Supporting this hypothesis, the results suggest that men evaluate women as romantic partners in facial attractiveness judgment by default and that men and women use different strategies in the attractiveness judgment of invisible faces of the opposite gender.

Recently, the number of people wearing sanitary masks has drastically increased due to COVID-19, and many Japanese people still wear masks in public areas. This has led them to guess the others’ full faces based on partial information (e.g., eye regions). Various studies have reported that wearing a mask hinders identification (Noyes et al., 2021), re-identification (Marini et al., 2021), and the recognition of others’ facial expressions (Gori et al., 2021; Grundmann et al., 2021; Roberson et al., 2012; Ruba & Pollak, 2020). Moreover, wearing masks tends to be rated as more attractive than not wearing them (Dudarev et al., 2022; Hies & Lewis, 2022; Kamatani et al., 2021; Patel et al., 2020). This is interpreted as the result of the physical occlusion of facial parts that are judged negatively (e.g., rough skin and acne) with masks.

Although no factors other than the effect of physical shielding have been considered for the heightened attractiveness of mask-wearing faces at present, we hypothesize that cognitive bias related to strategies of mate preference, as shown in Ichimura et al. (2021), is also responsible for it. The present study aimed to test this hypothesis and the robustness of gender difference in bias when perceiving the attractiveness of partial faces. Investigating cognitive bias in the attractiveness judgment of faces wearing masks is not only valuable from a practical perspective but also important for understanding the perception of partial views of faces.

## Experiment 1

We predicted that facial attractiveness would be modulated by assumed social relationships, which would show gender differences. More specifically, when rating women as romantic partners, men would rate women's faces with masks as more attractive than those without; yet, this tendency would be suppressed when rating women as friends. On the other hand, women's attractiveness judgment of male faces would be independent from assumed social relationships.

### Method

#### Participants

Based on a priori power analyses using G*Power (Faul et al., 2007), for the current factorial design, with a significance level of 0.025 with a Bonferroni correction (α = 0.05/2 = 0.025, see the Data Analysis section below) and a power of 0.80, along with a previously reported substantial effect size (*f *= 0.4), as observed in the study by Ichimura et al. (2021), a sample size of 12 participants per gender would be appropriate. To meet this requirement, we totally recruited 24 Japanese participants (12 women, mean age = 20.58 years) for this experiment. All participants had normal or corrected-to-normal vision and were unaware of the purpose of the study. All experiments were conducted in accordance with the principles of the Declaration of Helsinki. The study protocol was approved by the Ethics Committee of Chuo University (approval number: 2022-0076).

#### Apparatus

The stimuli were presented on a 23.8-inch display. Lab.js controlled the stimulus presentation and data collection (Henninger et al., 2022).

#### Stimuli

Photographs used as stimuli depicted shoulder-up color headshots of 40 Japanese models (20 women aged 21–23). Each model had a neutral expression and was photographed under two conditions: face wearing a white sanitary mask, the product of which was the same for all participants, and face without mask. Thus, the total number of stimulus images was 80. Photographs were controlled for distance, angle, and luminosity. Each photograph subtended 18.5° × 13.7° in the visual angle, in which the average size of the photographed head was about 8.5° × 7.5°. The viewing distance was about 40 cm.

**Figure 1.**
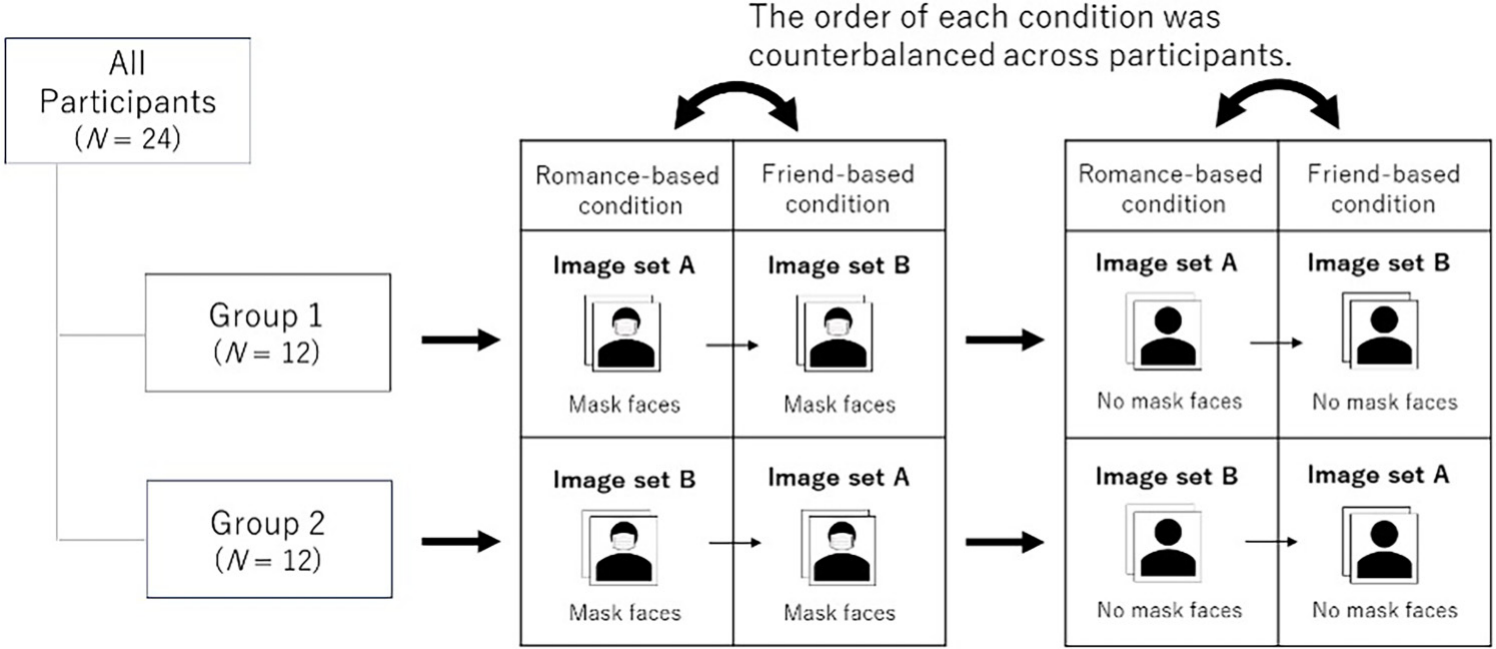
Summary of task manipulation.

The images of the 40 models were randomly divided into two sets (10 men and 10 women in set A and the others in set B). Half of the participants evaluated faces of set A as romantic partners (romance-based condition, see the procedure section for more details) and faces of set B as friends (friend-based condition, see the procedure section for more details); the other half evaluated set B under a romance-based condition and set A under a friend-based condition. Each participant viewed 20 mask images and 20 no-mask images of the same model in each set (80 images in total; Figure 1).

#### Procedure

In each trial, one photograph was randomly selected and displayed at the center of the screen. Participants were asked to rate the attractiveness of the displayed face on a 7-point scale (1 = *not attractive at all*, 7 = *very attractive*). Following the procedure used by Ichimura et al. (2021), all participants were instructed to rate the attractiveness of a person of the opposite gender as a romantic partner (romance-based condition) or as a friend (friend-based condition); a factor of social relationship was manipulated within participants but between blocks (Figure 1). In both conditions, participants were instructed to rate the attractiveness of a person of the same gender as a friend. The participants’ task remained the same under both the conditions as they evaluated a person of the same gender.

Each participant evaluated the faces wearing masks under the romance-based or friend-based condition in a separate block (20 trials in each block), followed by a block in which each participant evaluated the faces without masks under the romance-based or friend-based condition (20 trials in each block). The order of social relationships was counterbalanced across the participants.

#### Data Analysis

We subtracted the mean rating score for faces without masks from that for faces with masks for each participant, which was an index of the magnitude of mask bias. First, to investigate the occurrence of bias, we conducted one-sample *t* tests to determine whether the mask bias was significantly different from 0. Because we repeated *t* tests with the data of the opposite genders under the romance-based and friend-based conditions four times, the significance level was set as α = 0.05/4 = 0.0125 with a Bonferroni correction. Second, and more importantly, our prediction was set a priori based on Ichimura et al. (2021) and was clear; the mask bias for women's faces by male participants was smaller under the friend-based condition than under the romance-based condition. Therefore, the results of our interest were the differences in mask bias for the opposite genders between the friend-based and romance-based conditions. We conducted one-tailed paired *t* tests twice, with a significance level of α = 0.05/2 = 0.025 with a Bonferroni correction.

### Results and Discussion

**Figure 2.**
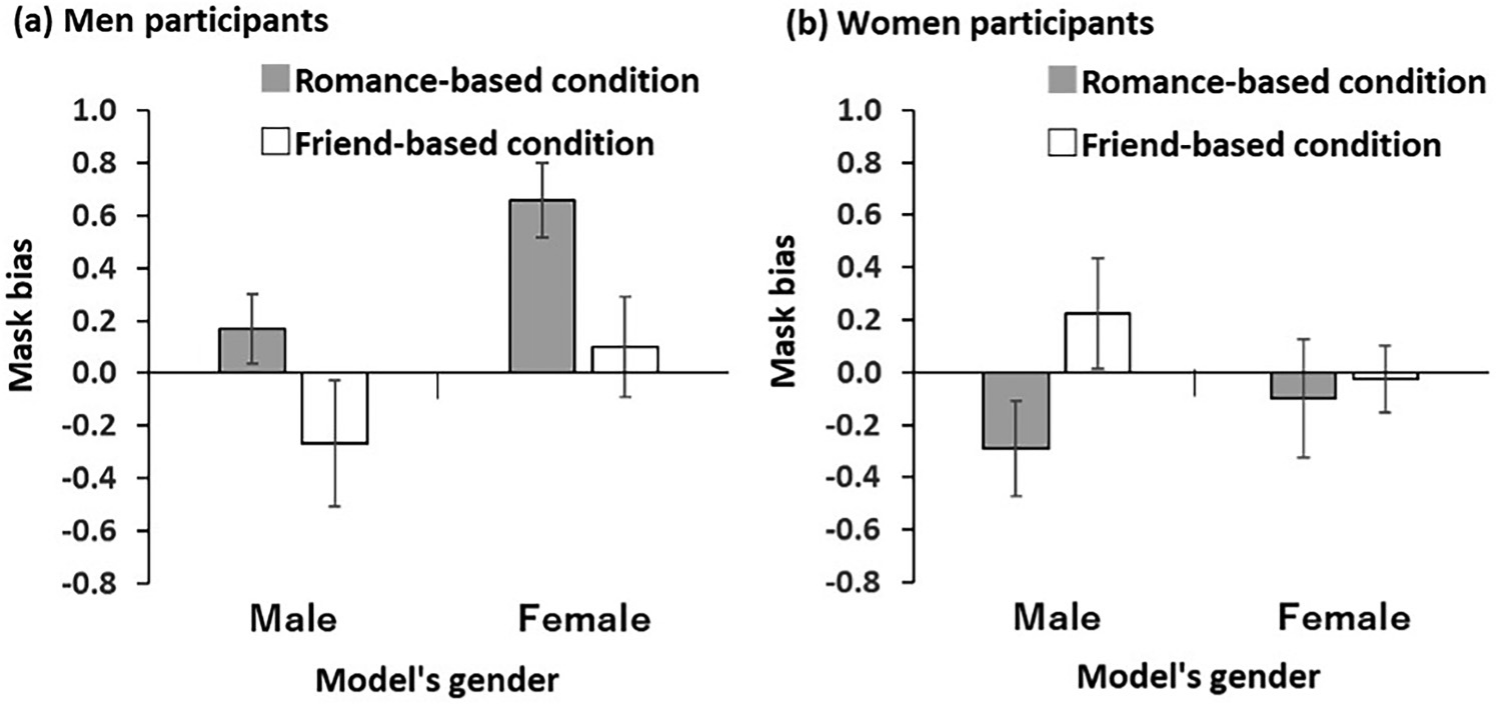
Results of Experiment 1. Error bars indicate the standard error of the mean.

Table 1 and Figure 2 show the descriptive statistics and the mean magnitudes of mask bias by male and female raters for the female and male models under the romance-based and friend-based conditions. The mask bias toward women's faces by men under the romance-based condition was significantly larger than zero, *t*(11) = 4.647, *p *< .05/4, Cohen's *d *= 1.340. The other mask biases toward the opposite genders were not significantly different from zero, *t*s(11) < 1.620, *p*s > .134, Cohen's *d*s < 0.467. Importantly, mask bias was significantly smaller when men rated women's faces under the friend-based condition than when they rated them under the romance-based condition, *t*(11) = 3.048, *p *< .05/2, Cohen's *d *= 0.880. However, when female participants rated men's faces, mask bias was significantly higher under the friend-based condition than under the romance-based condition, *t*(11) = 2.222, *p *< .05/2, Cohen's *d *= 0.641.

**Table 1.** The descriptive statistical values on the attractiveness of the opposite-gender models in Experiment 1.

		Romance-based condition		Friend-based condition	
		Mask	No mask	Mask	No mask
Men raters	*M (SD)*	3.79 (1.04)	3.13 (1.15)	3.80 (1.09)	3.70 (1.05)
Women raters	*M (SD)*	2.89 (1.18)	3.18 (1.47)	4.00 (1.17)	3.78 (1.45)

Men rated women's faces wearing masks as more attractive than faces not wearing masks when rating women as romantic partners (mask bias); however, such bias disappeared when men rated women as friends, that is, the assumed social relationship determined the occurrence of mask bias in men. For women, the attractiveness judgment of male faces was independent of wearing masks. These patterns of mask bias were similar to those of back-view bias (Ichimura et al., 2021), demonstrating the robustness of the gender difference in bias in perceiving the attractiveness of partial views of faces.

Unexpectedly, the magnitude of mask bias for male faces by women was smaller under the romance-based condition than under the friend-based condition, although the bias was not significant. Furthermore, considering that the mask bias under the romance-based condition was less than zero, women tended to rate the attractiveness of male faces wearing masks as more negative than that of faces not wearing masks. This is likely because masks function as a priming for unhealthiness (Miyazaki & Kawahara, 2016). It would be natural for women to emphasize unhealthiness more for romantic partners than for friends in terms of reproduction.

The current findings supported our hypothesis that mate preference strategies related to the reproductive cost underlie the attractiveness judgment of faces wearing masks. To strengthen our claim that the assumed social relationship (i.e., the possibility of reproduction) modulates men's bias toward women, the next experiment tested the mask bias with elderly male participants, who are thought to have less reproductive function, and compared it to the bias by young male participants. Furthermore, participants in Experiment 2 evaluated the facial attractiveness of only one individual (woman) as a potential mate as in a usual daily situation.

## Experiment 2

### Method

#### Participants

We recruited 94 young (mean age = 19.36 years) and 226 elderly (mean age = 69.70 years) male participants. Since the experiment was conducted online, and anticipating potential dropout, especially among elderly participants, we enlarged the sample size compared to Experiment 1. Consequently, seven elderly participants were excluded.

#### Apparatus

The participants’ own devices (PCs, tablets, or smartphones) were used.

#### Stimuli

The photographs of one woman, which had elicited the greatest mask bias in Experiment 1, were used.

#### Procedure

The procedure was the same as in Experiment 1, except the participants evaluated the faces wearing the mask and not wearing the mask (a within-subject factor) of only one woman under the romance-based (50 young and 107 elderly males) or friend-based (44 young and 112 elderly males) condition (a between-subject factor) in an online experiment.

#### Data Analysis

We analyzed the data using the same approach as in Experiment 1.

### Results and Discussion

**Figure 3.**
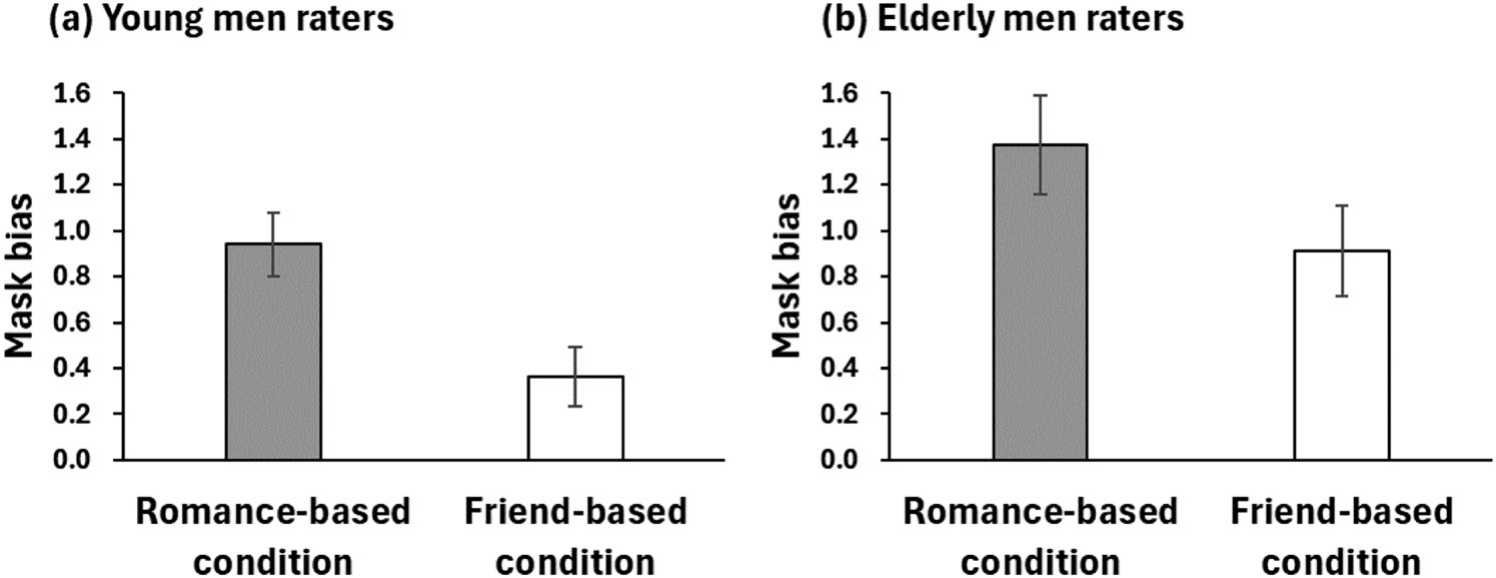
Results of Experiment 2. Error bars indicate the standard error of the mean.

Figure 3 shows the mean magnitudes of mask bias by young and elderly male raters for the female model under the romance-based and friend-based conditions. The mask biases by young men were significantly larger than zero under the romance-based, *t*(49) = 6.660, *p *< .05/4, Cohen's *d *= 0.942, and friend-based conditions, *t*(43) = 2.790, *p *< .05/4, Cohen's *d *= 0.364. The bias was significantly smaller under the friend-based than under the romance-based condition, *t*(92) = 2.970, *p *< .05/2, Cohen's *d *= 0.614. As for elderly men, the biases were significantly larger than zero under the romance-based, *t*(106) = 9.270, *p *< .05/4, Cohen's *d *= 0.896, and friend-based conditions, *t*(111) = 7.720, *p *< .05/4, Cohen's *d *= 0.729. The bias was significantly smaller under the friend-based than under the romance-based condition, *t*(217) = 2.460, *p *< .05/2, Cohen's *d *= 0.332.

First, the mask bias modulated in response to the assumed social relationship in Experiment 1 was successfully replicated by young men, demonstrating the robustness of the phenomenon. Second, unexpectedly, the elderly men also exhibited this bias, likely because they evaluate the facial attractiveness of young individuals higher than young participants (Foos & Clark, 2011; He et al., 2021), thus amplifying the bias. In fact, the effect size of the bias under the friend-based condition was larger for the elderly participants (Cohen's *d *= 0.729) compared to the young participants (Cohen's *d*s < 0.364 in Experiments 1 and 2). Importantly, the effect size of the assumed social relationship was smaller for the elderly participants (Cohen's *d *= 0.332) than for the young participants (Cohen's *d *= 0.880 in Experiment 1, Cohen's *d *= 0.614 in Experiment 2), though there was no significant interaction observed. Although the mask bias was observed for the elderly participants, it was less affected by the assumed social relationship likely due to reproductive function decline.

## General Discussion

We demonstrated that men rated women's faces hidden by masks as more attractive than unhidden faces when rating women as romantic partners; however, this bias disappeared when men rated women as friends (Experiment 1). In contrast, the attractiveness judgment of male faces by women was independent of wearing masks. The mask bias among the elderly male participants was less affected by the assumed social relationship (or the possibility of reproduction), compared to the young male participants, likely due to reproductive function decline, though the bias was observed (Experiment 2). These results generally support our hypothesis that cognitive bias related to reproduction underlies the attractiveness judgment of faces wearing masks.

The gender difference in back-view bias has been previously explained as follows (Ichimura et al., 2021). Men would overestimate the facial attractiveness of women as romantic partners by default to drive themselves to approach them by minimizing false-negative errors, or misses due to a lower cost of reproduction. On the other hand, women are motivated to minimize false-positive errors, or false alarms due to a higher cost of reproduction, which results in no need to overestimate men's facial attractiveness even though women rated them as romantic partners. Our findings are consistent with this hypothesis. Based on the hypothesis, the current young male participants might assume the short-term relationship in judging the romantic partner, whereas the female participants might assume the long-term relationship (Clark & Hatfield, 1989). That is, not only what is emphasized for the mate choice but also the assumed lengths of the partner relationship would vary with the reproductive cost, though they are inseparable. Given that individuals tend to overestimate the attractiveness of their romantic partners’ faces and body (e.g., Barelds et al., 2011; Swami et al., 2007), the overestimation of the physical attractiveness of the opposite gender, which is particularly observed for men, would be a robust and common cognitive strategy.

The current findings demonstrate not only the robustness of the bias reported in our earlier study (Ichimura et al., 2021) but also the possible role of the social relationship in the attractiveness judgment. Previous research demonstrated that the attractiveness ratings of the face with an exposure of 100 ms were positively correlated with those without a time limit, concluding that the facial attractiveness judgment is immediate (Willis & Todorov, 2006). However, the correlation was not perfect (*r *= .690), suggesting that the attractiveness judgment is modulated over time. In this respect, the current findings offer the possible cognitive process that the attractiveness formed immediately is vulnerable to the social relationship.

The current experimental paradigm favored the males’ strategy in the attractiveness judgment; precisely the focus of our study. It is crucial to demonstrate how people judge the facial attractiveness based only on visual stimuli, mirroring real-life encounters; for example, economic status is not usually provided in the first encounter. Therefore, the current finding specifically reflects the formation of the visual attractiveness of faces, rather than encompassing the entirety of mate choice.

In sum, the overestimation of facial attractiveness of the opposite gender, particularly for men, would be a common cognitive strategy of mate preference, which underlies the perception of partial faces. Men would automatically evaluate women using the criteria of romantic partners, but they could evaluate women as friends only when required.

## References

[bibr1-20416695241287486] Barelds D. P. H. Dijkstra P. Koudenburg N. Swami V. (2011). An assessment of positive illusions of the physical attractiveness of romantic partners. Journal of Social and Personal Relationships, 28, 706–719. 10.1177/0265407510385492

[bibr2-20416695241287486] Bateman A. J. (1948). Intra-sexual selection in Drosophila. Heredity, 2, 349–368. 10.1038/hdy.1948.21 18103134

[bibr3-20416695241287486] Clark R. D. Hatfield E. (1989). Gender differences in receptivity to sexual offers. Journal of Psychology & Human Sexuality, 2, 39–55. 10.1300/J056v02n01_04

[bibr4-20416695241287486] Dudarev V. Kamatani M. Miyazaki Y. Enns J. T. Kawahara J. I. (2022). The attractiveness of masked faces is influenced by race and mask attitudes. Frontiers in Psychology, 13, 864936. 10.3389/fpsyg.2022.864936 35656497 PMC9152543

[bibr5-20416695241287486] Faul F. Erdfelder E. Lang A. G. Buchner A. (2007). G* Power 3: A flexible statistical power analysis program for the social, behavioral, and biomedical sciences. Behavior Research Methods, 39, 175–191. 10.3758/BF03193146 17695343

[bibr6-20416695241287486] Foos P. W. Clark M. C. (2011). Adult age and gender differences in perceptions of facial attractiveness: Beauty is in the eye of the older beholder. Journal of Genetic Psychology, 172, 162–175. 10.1080/00221325.2010.526154 21675545

[bibr7-20416695241287486] Gangestad S. W. Scheyd G. J. (2005). The evolution of human physical attractiveness. Annual Review of Anthropology, 34, 523–548. 10.1146/annurev.anthro.33.070203.143733

[bibr8-20416695241287486] Gori M. Schiatti L. Amadeo M. B. (2021). Masking emotions: Face masks impair how we read emotions. Frontiers in Psychology, 12, 669432. 10.3389/fpsyg.2021.669432 34113297 PMC8185341

[bibr9-20416695241287486] Grundmann F. Epstude K. Scheibe S. (2021). Face masks reduce emotion-recognition accuracy and perceived closeness. PLOS ONE, 16, e0249792. 10.1371/journal.pone.0249792 PMC8064590 33891614

[bibr10-20416695241287486] Haselton M. G. Buss D. M. (2000). Error management theory: A new perspective on biases in cross-sex mind Reading. Journal of Personality and Social Psychology, 78, 81–91. 10.1037/0022-3514.78.1.81 10653507

[bibr11-20416695241287486] He D. Workman C. I. Kenett Y. N. He X. Chatterjee A. (2021). The effect of aging on facial attractiveness: An empirical and computational investigation. Acta Psychologica, 219, 103385. 10.1016/j.actpsy.2021.103385 34455180 PMC8438792

[bibr12-20416695241287486] Henninger F. Shevchenko Y. Mertens U. K. Kieslich P. J. Hilbig B. E. (2022). Lab.js: A free, open, online study builder. Behavior Research Methods, 54, 556–573. 10.3758/s13428-019-01283-5 34322854 PMC9046347

[bibr13-20416695241287486] Hies O. Lewis M. B. (2022). Beyond the beauty of occlusion: Medical masks increase facial attractiveness more than other face coverings. Cognitive Research: Principles and Implications, 7, 1–6. 10.1186/s41235-021-00351-9 35006366 PMC8743690

[bibr14-20416695241287486] Ichimura F. Moriwaki M. Ariga A. (2021). Romantic bias in judging the attractiveness of faces from the back. Journal of Nonverbal Behavior, 45, 339–350. 10.1007/s10919-021-00361-7

[bibr15-20416695241287486] Kamatani M. Ito M. Miyazaki Y. Kawahara J. I. (2021). Effects of masks worn to protect against COVID-19 on the perception of facial attractiveness. i-Perception, 12, 1–14. 10.1177/20416695211027920 PMC8243111 34262683

[bibr16-20416695241287486] Marini M. Ansani A. Paglieri F. Caruana F. Viola M. (2021). The impact of facemasks on emotion recognition, trust attribution and re-identification. Scientific Reports, 11, 1–14. 10.1038/s41598-021-84806-5 33692417 PMC7970937

[bibr17-20416695241287486] Miyazaki Y. Kawahara J. I. (2016). The sanitary-mask effect on perceived facial attractiveness. Japanese Psychological Research, 58, 261–272. 10.1111/jpr.12116

[bibr18-20416695241287486] Noyes E. Davis J. P. Petrov N. Gray K. L. H. Ritchie K. L. (2021). The effect of face masks and sunglasses on identity and expression recognition with super-recognizers and typical observers. Royal Society Open Science, 8, 201169. 10.1098/rsos.201169 33959312 PMC8074904

[bibr19-20416695241287486] Patel V. Mazzaferro D. M. Sarwer D. B. Bartlett S. P. (2020). Beauty and the mask. Plastic and Reconstructive Surgery Global Open, 8, e3048. 10.1097/GOX.0000000000003048 PMC7489620 32983796

[bibr20-20416695241287486] Perrett D. I. Lee K. J. Penton-Voak I. Rowland D. Yoshikawa S. Burt D. M. Akamatsu S. (1998). Effects of sexual dimorphism on facial attractiveness. Nature, 394, 884–887. 10.1038/29772 9732869

[bibr21-20416695241287486] Roberson D. Kikutani M. Döge P. Whitaker L. Majid A. (2012). Shades of emotion: What the addition of sunglasses or masks to faces reveals about the development of facial expression processing. Cognition, 125, 195–206. 10.1016/j.cognition.2012.06.018 22892280

[bibr22-20416695241287486] Ruba A. L. Pollak S. D. (2020). Children’s emotion inferences from masked faces: Implications for social interactions during COVID-19. PLoS ONE, 15, e0243708. 10.1371/journal.pone.0243708 PMC7757816 33362251

[bibr23-20416695241287486] Rule N. O. Ambady N. Adams R. B. (2009). Personality in perspective: Judgmental consistency across orientations of the face. Perception, 38, 1688–1699. 10.1068/p6384 20120266

[bibr24-20416695241287486] Swami V. Furnham A. Georgiades C. Pang L. (2007). Evaluating self and partner physical attractiveness. Body Image, 4, 97–101. 10.1016/j.bodyim.2006.10.003 18089256

[bibr25-20416695241287486] Thornhill R. Gangestad S. W. (1999). Facial attractiveness. Trends in Cognitive Sciences, 3, 452–460. 10.1016/S1364-6613(99)01403-5 10562724

[bibr26-20416695241287486] Willis J. Todorov A. (2006). First impressions: Making up your mind after a 100-ms exposure to a face. Psychological Science, 17, 592–598. 10.1111/j.1467-9280.2006.01750.x 16866745

[bibr27-20416695241287486] Yonemura K. Ono F. Watanabe K. (2013). Back view of beauty: A bias in attractiveness judgment. Perception, 42, 95–102. 10.1068/p7356 23678619

